# e-meducation.org: an open access medical education web portal

**DOI:** 10.1186/1472-6920-8-6

**Published:** 2008-01-24

**Authors:** Vangelis G Alexiou, Matthew E Falagas

**Affiliations:** 1 Alfa Institute of Biomedical Sciences (AIBS), Athens, Greece; 2 Department of Medicine, Tufts University School of Medicine, Boston, Massachusetts, USA

## Abstract

**Background:**

Internet can serve in opening the door to a brand new world of high quality medical information. However, the chaotic size of data available in the WWW is often misleading. We sought to provide the world medical community with a web portal that may be used as a clearinghouse providing the outlet for dissemination of high quality WWW educational products.

**Methods:**

Directories of the relevant WWW resources have been compiled and others are being currently under development to cover most medical fields. A custom-built medical search engine was created. Really Simple Syndication (RSS) feeds and video sharing services were reviewed for their quality and were presented along with case-based educational presentations through a user-friendly web portal interface. A directory of guidelines database is currently under development.

**Results:**

The educational portal "e-meducation" available at  has been launched in December 2006 and at the moment, provides links to more than 800 educational web-pages, more than 2100 clinical practice guidelines, 32 news feeds, and 14 educational videos. The web site also hosts 40 case-based presentations and a custom medical search engine.

**Conclusion:**

Based on the incorporation of simple and tested educational strategies such as case based instruction and interactive learning, e-meducation.org aims to become a prototype platform that offers a more convenient interface to existing products, resources and medical contents.

## Background

The use of the World Wide Web (WWW) had major impact on almost every aspect of human life and activity including medical education. Internet can serve in opening the door to a brand new world of high quality medical information. However, the chaotic size of data available on the WWW, estimated to almost 532 terabytes in 2002 [1] is often misleading.

Open access (OA) is a relatively new movement in medicine and science in general that came with the advent of Internet, bringing immediate, free and unrestricted online access to digital scholarly material [2]. Berlin Declaration on Open Access to Knowledge in the Sciences and Humanities [3] in October 2003 provided the current definition of OA. OA means free availability on the WWW with permition to read, download, copy, redistribute, print, and to any other legitimate use. OA does not have any financial, legal and technical limitations, however, on reproduction and redistribution authors should be properly acknowledged and cited [4].

The existing medical portals, although very helpful, are specialized for a certain field (Portal of Geriatric Online Education [5], Family Medicine Digital Resources Library [6] and/or are libraries of copyrighted material that owners upload and share under specific terms and conditions [7]. Merlot is an educational portal created by the academic community to host educational products. However, it is multidisciplinary (various scientific fields) and limited to academic content. Ron LaPorte's SuperCourse [8] is a library of lectures created by faculty members from all over the world.

We sought to provide the world medical community with a web portal that will index, with an efficient and scientific way, quality OA medical education material available over the Internet and will serve as a tracking and filtering tool. A clearinghouse that may provide an outlet for dissemination of high quality WWW educational products and will become an online "bookmark folder" for healthcare professionals who wish to share with colleagues their favourite medical websites and discover several more.

## Methods

We sought to track web sites providing educational resources in 14 medical fields by making use of popular Internet search-engines (Google, AltaVista, and Yahoo). We used various relevant keywords in different combinations in our searches and reviewed the web pages found in depth of the first 2000 results. We also performed searches of the PubMed and Current Contents databases in our attempt to identify relevant electronic sources. In addition, we reviewed the information provided in web sites of major universities and institutions.

We chose to include in our indexes English language WWW resources, which are updated in a regular basis and have been active for more than a year. Criterion for inclusion of a potentially relevant WWW site was compliance with the OA movement principles. We excluded sites offering original research such as medical journals with OA, which are already known and easily accessible to the large medical community. To assure the integrity and quality as well as increase the accessibility of our web research output, we submitted the produced results in journals relevant to each of the fields, for peer review. Some of this work has already been published [9-15] and the rest is under consideration for publication in peer-reviewed biomedical journals of the relevant fields.

Another major source of online medical education material is video sharing services such as youtube and google video. Videos are freely distributed and available to view download and redistribute, in compliance with OA. Following the principles described above we sought to review categorize and present them along with a short description, interesting remarks, teaching points and relevant web links.

Internet has been revolutionised by the search engine technology presented by Google in September 1998. We used the co-op service [16] to create a custom medical search engine integrating Google's core search technology and restricting search results based on websites and pages selected by the process described above.

Really Simple Syndication (RSS) is a web feed format used to publish frequently updated digital content, such as journals scientific articles and news feeds [17]. We compiled a list of important medical RSS feeds grouped into categories permitting the user to review in a glance tens of major medical websites for news headlines, abstracts and links to the full articles and immediately retrieve the content he is interested in.

Furthermore, we created our own section of case based education. The chief complaint, history of present illness, medical history, physical examination, and diagnostic work up are presented in a succinct way leading to a question for the reader about diagnosis or management. Finally, we developed a database of international evidence-based clinical guidelines issued by international or national medical specialty associations, relevant professional societies and government agencies. Guidelines are browsable by medical specialty or through the search engine feature that queries for certain keyword.

In order to assure the reliability and credibility of the information published in e-meducation.org we applied and received the accreditation of "trustworthy health and medical information" issued by the Health On the Net Foundation (HON), a non-profit organisation that has elaborated a Code of Conduct to help standardise the reliability of medical and health information available on the WWW [18].

We chose not to include in this manuscript several technical details regarding the development of the website. However, a detailed technical report is available online [19].

## Results

The web-portal is available online [20]. The top horizontal navigation menu includes content informative and identifying over the objectives and aims, financial disclosure, advertising policy and authoritative of e-meducation.org. The privacy protection policy states the confidentiality of data relating to individual patients and visitors of e-meducation and the "contact us" link, provides information in the clearest possible manner about the persons responsible for the website, contact addresses and e-mail addresses, for visitors to seek further information or support. Under the link "AIBS teaching cases" [21] the visitor will find a selection of 40 case-based educational presentations offered by the clinicians of AIBS. A new case is added at least every month. The guidelines database is under construction and has been so far populated with 2100 guidance papers issued by major scientific societies [22]. Finally under the link "RSS feeds" we have put a selection of 32 RSS feeds grouped in 5 categories; continuing medical education, medicine in general, general content medical journals, infectious diseases and surgical journals [23].

In the left vertical navigation menu under the title "OA education", appear the compiled indexes of OA medical education resources [24]. In total we present more than 800 educational WWW pages grouped in 14 categories; internal medicine, infectious diseases, antimicrobial resistance, nosocomial infections, Hepatitis B virus, dermatology, orthopaedic infections, obstetrics and gynaecological infections, surgical infections, urogenital infections, cardiovascular infections, upper and lower respiratory infections and critical care and sepsis. The links are accompanied by a small description and comment over the presented webpage.

Registered users can add relevant links to the list in order to enrich this directory; the link becomes active once reviewed by the administrator of e-meducation. In the same navigation menu, under the title medical video resources, we index quality medical videos retrieved from video sharing services. At the time, only laparoscopic video resources and physical examination videos are presented [25,26]. Below this menu the user can find the custom medical search engine, the teaching cases RSS feed and the login and registration form. Registration is complementary, free and not essential to browse through e-meducation content. Registered users receive newsletters with updates of the e-meducation content and can contribute by adding links to the compiled resources directory.

## Discussion

E-meducation is an effort to systematically index OA medical education material available on the Internet. However it is not yet complete in terms of content and definitely presents several limitations. The resources listed in this portal are far from exhaustive and the methodology used permitted us to retrieve only a certain subset of the relevant WWW resources. However, the interactive form of this portal permits end-users to enrich the compiled directories and add possibly omitted by the authors, websites. It should also be mentioned that the quality of the listed resources is not uniform and varies considerably. Another major limitation is that at the time, e-meducation only covers a small proportion of the various medical fields and disciplines. Nevertheless we are constantly devoting time and human resources to update the web portal with more and better medical education content. The major advantage of Internet is the dynamic form of content. However, it is at the same time, a drawback, as pages and resources often change addresses resulting in "dead" links and lost references. We need to face this by constantly monitoring our links for their integrity.

We are confident that despite the limitations and shortcomings, e-meducation can contribute from a new perspective to the OA movement. This project is based on the incorporation of simple and tested medical education strategies such as case based instruction and interactive learning, with the potential that Web technology offers and the principles of unrestricted use, distribution, and reproduction. It aims to become a prototype platform that offers a more convenient interface to existing products, resources and medical contents that are already freely available over the net, but are not exploited to their full potential.

## Competing interests

e-meducation.org is the educational portal of the Alfa Institute of Biomedical Sciences (AIBS). MEF is the director of AIBS; VGA is member of the educational committee of AIBS and administrator of e-meducation.org.

## Authors' contributions

Both VGA and MEF contributed to the idea of developing e-meducation.org and the preparation of this article. All authors read and approved the final manuscript.

## Pre-publication history

The pre-publication history for this paper can be accessed here:


